# Knowledge, attitude and practices of environmental health practitioners conducting food-borne disease outbreak investigation at a local municipality in Gauteng province, South Africa

**DOI:** 10.4102/hsag.v25i0.1359

**Published:** 2020-06-29

**Authors:** Thokozani P. Mbonane, Nisha Naicker

**Affiliations:** 1 Department of Environmental Health, Faculty of Health Sciences, University of Johannesburg, Johannesburg, South Africa; 2 School of Public Health, Faculty of Health Sciences, University of the Witwatersrand, Johannesburg, South Africa; 3 Epidemiology and Surveillance Section, National Institute for Occupational Health, Johannesburg, South Africa

**Keywords:** knowledge, attitude, practices, perceptions, food-borne disease, outbreaks and environmental health

## Abstract

**Background:**

Food-borne disease (FBD) outbreaks are a common occurrence that is either not investigated or poorly investigated. According to anecdote evidence, this is because of non-uniformity to environmental health practices in South Africa.

**Aim:**

This study aimed to determine and describe the knowledge, attitude and practices (KAP) of environmental health practitioners (EHPs) when conducting outbreak investigations of FBD at a local municipality.

**Setting:**

This study was conducted in three sub-districts of Ekurhuleni Metropolitan Municipality (EMM), one of five municipalities in Gauteng province, South Africa.

**Methods:**

A descriptive cross-sectional study was conducted to collect data using a semi-structured questionnaire. Data collected were analysed using IBM Statistical Package for the Social Sciences. Continuous variables were compared using analysis of variances, and correlation was used to determine any association.

**Results:**

Knowledge responses were scored out of 9. Open-ended questions were themed into five items (support, guidelines, resources, training and specialisation). Sixty-one (76.3%) participants were randomly selected to participate in the study. There were more female participants (55.7%) than male participants, and the mean age was 30.9 years. The participants’ knowledge scores ranged from 1 to 9. There were 17 (27.9%) participants who have conducted FBD outbreak investigation. Twenty-six (42.6%) believed that they were properly trained to conduct FBD outbreak investigations. Age was associated with position (*p* = 0.000) and qualification (*p* = 0.033).

**Conclusion:**

The results indicated that there are gaps and challenges in the knowledge, while the practices were not consistent amongst environmental health practitioners. However, the attitude of EHPs was positive with regard to their role in FBD outbreak investigations.

## Background

Food-borne disease (FBD) outbreaks are a growing public health concern in developing countries, and are associated with high morbidity and mortality (Havelaar et al. 2015; Scott 2003). Food-borne diseases are linked to the consumption of food or water contaminated with toxins, viruses, bacteria or parasites, while FBDs occur where more than two persons have acquired the disease from the same contaminated food eaten at the same time (Dewey-Mattia et al. 2018; Jones et al. 2004). The occurrence of food-borne outbreaks is common, and the magnitude can be local, nationwide and international, thus requiring resources at all levels as well as numerous skills and knowledge (e.g. environmental health) to investigate (Ammon & Tauxe 2007; Bélanger et al. 2015; Bisholo, Ghuman & Haffejee 2018; Niehaus et al. 2011; Skovgaard 2009).

The prevalence of FBD and outbreaks is well known in developed countries compared to developing countries, where a lack of reporting is a major issue (Rocourt et al. 2003). The severity of FBD and outbreaks is relatively higher in developing and underdeveloped countries because of socio-economic and environmental factors (Broner et al. 2010; Newman et al. 2015). The populations at risk and those most likely to have a high mortality rate are infants, young children, the ageing and immunocompromised individuals (Rocourt 2013). Globally, it is estimated that 420 000 deaths are associated with FBDs each year, while approximately 91 million persons get sick annually – 25 000 being children under the age of 5 years (World Health Organization 2015).

Numerous FBD outbreaks are suspected to be poorly investigated and in some instances not investigated (World Health Organization 2008), especially in low- or middle-income countries because of the non-existence or poor implementation of surveillance programmes (Käferstein 2003). A qualitative study on the practices of environmental health specialists (refer to as environmental health practitioners [EHPs] in South Africa) during FBD outbreak investigation showed that practices amongst environmental health specialists were not consistent, and were hindered by numerous organisational and operational factors (Selman & Green 2008). These included a lack of outbreak investigation training and experience, inadequate staff members, minimum or non-existent management support and poor collaboration between agencies (Selman & Green 2008). These factors may lead to a poor outbreak investigation with the possibility of not identifying the environmental factors that contributed to the occurrence of an outbreak. Environmental health investigations can also be hindered by different factors such as unpreparedness, non-compliance with sampling procedure, poor reporting and delays in reporting (Department of Health Directorate: Food Control 2000).

In South Africa, a number of FBDs have occurred, and were reported and investigated in the past. However, the role of EHPs was not clearly defined in these reports (Shonhiwa et al. 2019). The environmental health scope of practice states that the role of an EHP is to identify environmental factors that could have contributed to the outbreaks (Coleman 2016; National Department of Health (NDoH), 2009). An environmental health investigation includes environmental, food and water sampling and traceback investigation. Furthermore, an EHP should collect environmental samples of all utensils that could have been used during the preparation and serving of the food implicated (Department of Health Directorate: Food Control 2000). Food samples are not always available, as either the food could have been all served or leftovers disposed of. In such a case, EHPs should review the production and distribution chain of the suspected food (Weiser et al. 2016). According to anecdotal and scientific evidence in South Africa, laboratory and epidemiological investigations are well organised, conducted and reported compared to environmental health investigations (Muvhali et al. 2017; Ntshiqa et al. 2016; Smith et al. 2019; Sigudu, Tint & Archer 2015). Furthermore, little has been written about the actual procedures followed during environmental health investigations in South Africa, except guidelines written in 2010 (Department of Health Directorate: Food Control 2000). The aim of the study was to determine and describe the knowledge, attitudes and practices of EHPs concerning investigations of FBD outbreaks at a metropolitan municipality in Gauteng province, South Africa.

## Research methods and design

### Study design, site and population

**FIGURE 1:**
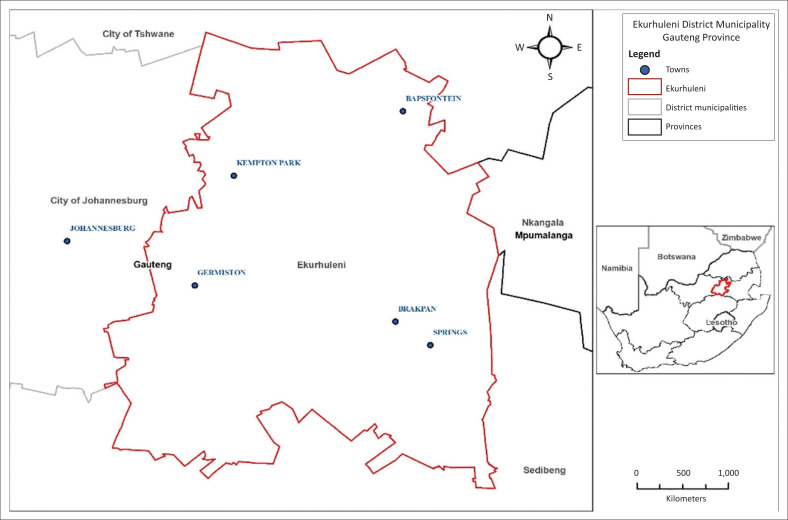
Map showing main towns of the subdistricts.

A descriptive cross-sectional study was conducted amongst EHPs registered with the Health Professions Council of South Africa (HPCSA) and employed at Ekurhuleni Metropolitan Municipality (EMM), one of five municipalities in Gauteng province, South Africa. The EHPs are stationed in three subdistrict regions (SDRs) of the municipality, namely, South SDR (Germiston), North SDR (Kempton Park) and East SDR (Springs), as shown in Figure 1. Each EHP at municipalities is responsible for rendering municipal health services (MHS) within their area of jurisdiction. These services include water quality monitoring, food control, waste management, health surveillance of premises, surveillance and prevention of communicable diseases (excluding immunisations), vector control, environmental pollution control, disposal of the dead and chemical safety (Balfour 2006). The researchers received permission from the Director of Municipal Health Services to access the list of EHPs in the municipality, and to approach them. At the time of conducting the study, there were 103 permanently employed EHPs, including supervisors and SDR managers servicing a population of 3 178 470 million (Municipalities of South Africa 2019). The sample size of 80 was calculated using a sample size calculator with a 95% confident interval, and a 5% error of margin for a known population. The EHPs were then grouped according to their SDR, and randomly selected. The structured questionnaires were sent to the area manager secretary’s office for distribution. A total of 61 EHPs (response rate of 76%) from 80 potential participants completed and returned the questionnaire.

### Data collection

A semi-structured questionnaire was used to collect information on demographic data of the participants, knowledge on conducting food-borne outbreak investigations, attitude on conducting and improving the investigations, and current practice of EHPs when conducting outbreak investigations in the municipality. The participants were further asked to give recommendations to improve outbreak investigations. The questionnaire was designed in English, as the participants were deemed conversant because of their educational level required to register with the HPCSA as independent EHPs. Questionnaires were handed and distributed by the secretary of the each SDR manager, and were collected after 7 days. The questionnaire was developed using the literature, as there were no previous studies conducted on the topic at the time the study was conducted (as per the knowledge of the researcher). Furthermore, it was pre-tested prior to the actual study to ensure accuracy and reliability. Ten EHPs from the three SDRs were invited to partake in the pilot study, and they were excluded from the actual study.

### Data management and analysis

Data from questionnaires were entered and captured on Microsoft Excel for cleaning and sorting. Thereafter, data were transferred to IBM Statistical Package for the Social Sciences (SPSS) Statistics Version 25 for analysis. Participants were given one point for a correct response in the knowledge section, whose points were then totalled. The data on the other variables (attitude and practices) of interest were not scored. Responses on the recommendation were themed into four categories after participants responded: guidelines, resources, training and notification. Descriptive analyses such as frequency, mean, standard deviation and range were conducted. Analysis of variance (ANOVA) was used to compare means of continuous variables (demographic information). The association between knowledge score and demographic information was determined using correlation. The level of significance was set at 0.05.

### Ethical consideration

Ethical approval for the study was granted by the Faculty of Health Sciences’ Academics Ethics Committee of the University of the Johannesburg. Permission to conduct the study was secured from the EMM. Participants were given an information sheet describing the aim and objectives of the study and signed written consent prior to taking part in the study. Furthermore, they were informed of voluntary participation, and the right to withdraw anytime without any punitive action. The ethics approval number for the study was AEC75-01-2013. Ethical clearance was sought from and granted by the University of Johannesburg, Faculty of Health Sciences, Research Ethics Committee (Ethical Clearance Number: AEC75-01-2013).

## Results

From the 61 (76.3% response rate) EHPs who volunteered to participate in the study, 34 (55.7%) were female participants and 27 were male participants. Amongst these, 37.7% (*n* = 23) were from South SDR, 34.4% (*n* = 21) were stationed at East SDR and 27.9 (*n* = 17) were from North SDR. Participants held different positions, and the majority were at an operational level (73.8%). The highest formal qualification obtained by one participant was a master’s degree, 32 (52.5%) participants had a BTech degree and 21 (34.4%) participants had a national diploma, while others (4.9%) had either an advanced diploma or honours degree in another field as an additional qualification (see more details on Table 1). The participants’ age ranged from 23 to 53 years, while the mean (± SD) was 30.9 (± 7.8) years.

**TABLE 1:** Demographic information of environmental health practitioners.

Demographic information	Frequency (no.)	Percentage
**Gender**		
Male	27	44.3
Female	34	55.7
**Age**		
21–25 years	14	23.0
26–30 years	28	45.9
31–35 years	7	11.5
36–40 years	4	6.6
41–45 years	2	3.3
46 or older	6	11.5
**Subdistrict station or office**		
East	21	34.4
South	23	37.7
North	17	27.9
**Position**		
EHP	45	73.8
Senior EHP	14	23.0
Assistant Chief EHP	2	3.3
**Years of experience**		
0–5 years	35	57.4
6–10 years	14	23.0
11–15 years	3	4.9
16 years above	9	14.8
**Highest formal qualification**		
National diploma	21	34.4
Advanced diploma	4	6.6
BTech degree	32	52.5
Masters	1	1.6
Others	3	4.9

EHP, environmental health practitioner.

There was no statistical significance between the EHPs from the three subdistricts for gender (*p* = 0.451), age (*p* = 0.595), position (*p* = 0.200), years of experience (*p* = 0.733) and highest formal qualification (*p* = 0.378). Hence, the data were merged for further analysis (for correlation). In the merged data set using Pearson’s correlation, a positive significant relationship between age and position (*r* = 0.544; *p* = 0.000) was found. Furthermore, there was a positive significant relationship between age and qualification (*r* = 0.237; *p* = 0.033).

### Knowledge of food-borne disease outbreak investigations

There were nine questions on knowledge relating to FBD outbreak investigations (see Table 2 for the detailed findings on knowledge). Participants were asked to define ‘food-borne disease outbreak’. Fifteen responded (24.6%) provided the correct response, namely, that FBD outbreak is the occurrence of two or more cases of FBDs from the ingestion of a common food at the same time and place (Dewey-Mattia et al. 2018). Nine (14.8%) gave a correct answer on the importance of conducting an environmental investigation (i.e. finding the environmental factors that contributed to the occurrence of FBD outbreaks and prevent future outbreak of similar nature). Only 10 (16.4%) participants gave a correct response (interview those involved, take food and environmental samples, and environmental assessment of the facility at the site where an outbreak has occurred).

**TABLE 2:** Participants’ responses to the knowledge questions.

Question	Correct (%)	Incorrect (%)
How would you define a food-borne diseases outbreak?	24.6	75.4
What is the difference between food-borne disease and food-borne diseases outbreak?	13.1	86.9
Why is it important to carry out an immediate environmental health field investigation?	14.8	85.2
What would be your actions at a food-borne disease outbreak site?	16.4	83.6
What is the temperature at which food samples should be kept at?	18	82
How would you ensure that the chain of custody is maintained after a food sample has been collected?	31.1	68.9
What is a food traceback investigation?	52.5	47.5
What type of samples will you take during a food-borne disease outbreak?	55.7	44.3
What is the recommended time frame to complete and submit a report on food-borne disease according to current legislation?	29.5	70.5

Questions on the adequate temperature (−2^°^C) at which samples must be kept were answered correctly by 11 (18.0) participants, compared to 50 (82.0) incorrect answers received. Nineteen (31.1%) participants knew how to maintain a chain of custody (ensuring that there is evidence or a paper trail of handling samples safely from the source to the laboratory). Approximately half (*n* = 32; 52.5) of the participants knew how to conduct a food traceback investigation. Thirty-four (55.7%) EHPs knew which type of samples (food and environmental, excluding stools) to collect during an investigation of food-related disease outbreak. The score out of the total (Skovgaard 2009) on the participants’ FBD outbreak knowledge ranged from 1 to 9, with a mean (± SD) of 2.4 (± 1.5) points. There was no correlation between the knowledge score with any demographic items.

### Environmental health practitioners’ attitudes towards food-borne disease outbreaks

**FIGURE 2:**
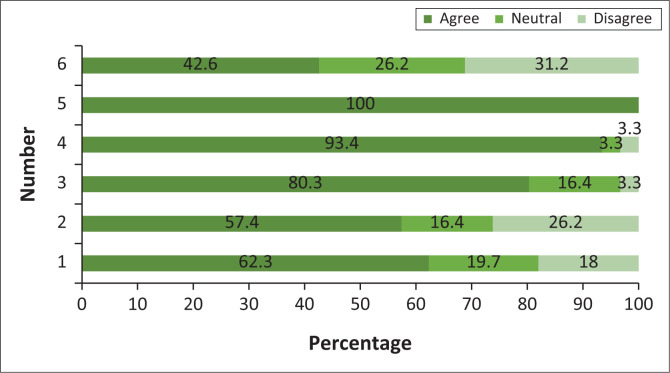
Environmental health practitioners’ attitudes towards outbreak investigations.

Twenty-six (42.6%) participants believed that they were properly trained to conduct FBD outbreaks. All participants agreed that EHPs have a role in reducing the spread of food poisoning cases. Fifty-seven (93.4%) participants believed that environmental health investigation has an impact on an outbreak investigation. Most participants (*n* = 49; 80.3%) indicated that it is important to conduct follow-up and monitoring visits after an outbreak. More than half (*n* = 35; 57.4%) of the participants indicated that it is difficult to conduct investigations and follow-ups effectively while trying to meet all the functions of MHS. Thirty-eight (62.3%) participants believed that specialising in environmental epidemiology is necessary for conducting an effective outbreak investigation. A detailed description of participants’ attitudes is provided in Figure 2.

Using Pearson’s correlation, a relationship between the statement ‘I was properly trained to conduct investigations’ was found with age (*r* = 0.262; *p* = 0.041) and years of experience (*r* = 0.324; *p* = 0.011). This showed that older participants and long-serving EHPs believed that they were properly trained.

### Practices of outbreak investigations

Feedback on the participants’ current practices when conducting outbreak investigations is summarised in Table 3. Seventeen (27.9%) participants indicated that they have conducted an FBD outbreak investigation, compared to 44 (72.1%). Forty (65.6%) participants stated that they knew about an occurrence of an outbreak immediately when it had occurred. Thirteen (21.3%) respondents stated that they knew about an outbreak a day after it has occurred, while five (8.2%) participants knew after evidence (suspected food) was removed. Twenty-eight (45.9%) participants took samples within the first 12 h after the outbreak has occurred, while 33 (54.1%) participants took samples after 12 h. The majority (91.8%) of the participants submitted samples to the laboratory within 12 h after collecting them. More than half (*n* = 31; 50.8%) of the participants ‘always’ conducted follow-up or monitoring visits, and 21 (34.4%) EHPs sometimes conducted these visits and nine (14.8%) never conducted monitoring visits. Out of the 52 participants who either always or sometimes conducted follow-up visits, 20 (38.5%) took 2 weeks to conduct these visits, 13 (25.0%) a week, 12 (23.1%) a month and seven (13.5%) took more than a month. Approximately 20% reviewed and requested documents (e.g. notification letters), 17 (27.9%) called a relevant person and 52.5% either consulted their supervisor or colleagues. Six (9.8%) EHPs prepared for outbreak investigation by arranging sampling equipment and liaised with the outbreak response team (ORT), while 11 (18.0%) and 26 (42.6%) only communicated with ORT and arranged sampling, respectively.

**TABLE 3:** Practices in relation to outbreak investigations.

Questions	Responses	Frequency (no.)	Percentage (%)
Have you been involved in a food-borne disease outbreak investigation as an EHP?	No	44	72.1
	Yes	17	27.9
When do you become aware of food-borne diseases outbreak?	Immediately when it has occurred	40	65.6
	A day after it has occurred	13	21.3
	When all evidence has been removed	5	8.2
	Other	3	4.9
While in the office, how would you confirm or determine that an outbreak may have occurred?	Reviewing and requesting documents	12	19.7
	Calling relevant employees	17	27.9
	Other	32	52.5
How do you prepare for an outbreak investigation before going out?	Arrange sampling equipment	26	42.6
	Communicate with ORT and arrange sampling equipment	6	9.8
	Communicate with ORT	11	18.0
	Other	18	29.5
What is the appropriate time frame for taking a sample after an outbreak has occurred?	Last 12 h	28	45.9
	Last 24 h	18	29.5
	Last 48 h	4	6.6
	Last 72 h	3	4.9
	Other	8	13.1
After collecting the food sample, how long on average does it take you to submit the samples to the laboratory?	Within 12 h	56	91.8
	Between 12 and 24 h	5	8.2
How often do you conduct follow-up or monitoring visits after report writing?	Never	9	14.8
	Sometimes	21	34.4
	Always	31	50.8
How long after the investigations do you conduct a follow-up or monitoring visit?	One week	13	25.0
	Two weeks	20	38.5
	One month	12	23.1
	More than 1 month	7	13.5

ORT, outbreak response team; EHP, environmental health practitioner.

The results differed from the guidelines developed in 2010, which stipulates that EHPs should be informed immediately after an outbreak has occurred (Department of Health Directorate: Food Control 2000). This will allow them to confirm the occurrence of the outbreak by contacting the reporting facility and reviewing notifications. While in the office, they need to prepare for the investigation through gathering of materials needed and contacting the ORT if a need arises. Samples of the suspected food should be collected immediately and submitted to the laboratory for analysis. Furthermore, it is expected that EHPs conduct a follow-up visit to monitor implementation of the investigation’s recommendations as per the guidelines (Department of Health Directorate: Food Control 2000).

Sixty (98.4%) participants recommended that the municipality should develop a guideline document in a form of standard of the procedure (SOP). Approximately 69% of participants indicated the need to provide adequate training in environmental epidemiology, and 57.4% (*n* = 35) suggested that the municipality should introduce specialisation in the rendering of MHS. All the participants (*n* = 61; 100%) mentioned the provision of adequate resources to conduct a successful outbreak investigation, as well as a need for management support.

## Discussion

This study demonstrated that there is inconsistency in terms of knowledge and practice of EHPs pertaining to outbreak investigations. According to Shonhiwa et al. (2019), there is a large degree of inconsistency in the reporting and investigation of FBDs throughout South Africa (Shonhiwa et al. 2019). However, this is not localised to South Africa. In the United States, only 57% of reported food-borne-related outbreaks were investigated properly (Krosnick 1999), and hence, the importance of determining the KAP on FBD environmental outbreak investigations amongst EHPs. This is the first study to determine KAP on FBDs outbreak investigation amongst EHPs in South Africa. The knowledge on outbreak investigations concepts was low. However, the attitude was positive and the practices were inconsistent. The EHP per population ratio was 1:30 859. This is too high when compared to the World Health Organization’s recommendation (1 EHP: 10 000 population) and the South African adopted standard of 1 EHP: 15 000 population (Balfour 2006). This could obstruct the provision of Environmental Health Services (EHS) and its programmes – especially FBDs outbreak investigation – as highlighted by Shezi and co-authors (Shezi et al. 2019).

The knowledge score on FBDs and its outbreak investigations were poor. This was similar to other studies conducted amongst public health officials (Mathatha et al. 2018). The number of correct responses was very low (88.6% scored less than four out of nine), and respondents responded as follows: differentiating between FBD and FBD outbreak (13.1%), importance of conducting outbreak investigation (14.8%), activities at the outbreak site (16.4%), maintaining the cold chain (18.0%), chain of custody (31.1%) and time frame to finalise and provide feedback (29.5%). This could impair on the urgency of conducting an outbreak investigation, as these differ from routine inspections (Petran, White & Hedberg 2012). Such conduct might lead to failure in determining the environmental factors that contributed to the occurrence of an outbreak, and may have detrimental consequences such as failure to prevent further outbreaks.

Even though most participants had a BTech degree, more than 50% believed that they were not properly trained, or were not sure. This was mostly influence by age (*p =* 0.041) and the number of years as a practicing EHP (*p =* 0 011*).* However, participants had a positive attitude towards their role in outbreak investigation and the impact of their investigations. This could mean that EHPs are willing to be trained to improve their skills and to contribute meaningfully to the outcomes of outbreak investigations. Previously, the environmental health curriculum was not similar in South African institutions that offered the course, especially at BTech degree level, where students branched to specialisation. This could have created this knowledge gap amongst EHPs. The recently approved and implemented curriculum sought to address that challenge. This intervention will, however, not assist EHPs who are already in the field. In this study, EHPs believed that performing all nine MHS hinders them from being effective and conducting follow-up visits. However, they believed that specialisation in the profession is a solution to this challenge. There is a need to investigate this further, as most health professions have specialised in rendering health services.

In South Africa, there are a few outbreaks related to food contamination reported. Environmental practitioners rely on the clinical or laboratory staff for FBD outbreak notification. This could be explained by the high number of EHPs (72.1%) who had not conducted an outbreak investigation prior to taking part in the study. According to Bisholo and colleagues, South Africa does not have the capacity to stop and prevent FBD outbreaks. In addition, lack of standardisation and uniformity in the provision of EHS have been a concern for a long time in South Africa (Balfour 2006), hence the development and implementation of the National Environmental Health Policy in 2013 (Department of Health 2014). Out of the seven questions on the practice, none of the activities showed consistency amongst EHPs. These findings are similar to a study in 2008 which found significant dissimilarity in the practice amongst environmental health specialists conducting outbreak investigations (Selman & Green 2008).

The participants recommended the provision of adequate resources for conducting outbreak investigations. This has been an ongoing challenge that was previously raised by other officials and researchers (Balfour 2006; Department of Health 2014; Department of Health Directorate: Food Control 2000; Nahman, Wise & De Lange 2009; Wright, Mathee & Oosthuizen 2013). Environmental health practitioners suggested the development of guidelines in a form of SOP on outbreak investigation that will be compulsory to follow; yet there are national guidelines established in 2010 (Department of Health Directorate: Food Control 2000). This could mean a low level of awareness about this document, or lack thereof amongst EHPs. Encouragingly, they highlighted a need for specialised training on environmental health outbreak investigation. Mathee and Wright (2013) believed that specialised training can improve special skills in EHS (Mathee & Wright 2013), as EHPs are trained on an array of fields of public health. Furthermore, they called for specialisation within the EH profession, as most believed that this will enable EHPs to become experts, rather than trying to achieve all functions of EHS at once. They also highlighted a need for management support in EHS provision. Some of these recommendations (management support, specialised training and provision of resources) are similar to those highlighted in another study on other functions of EHS (Shezi et al. 2019).

Municipal health services are part of primary health care at local government level (Robertson 2016). One of the major roles of EHPs at local government level is to monitor, control and manage environmental factors contributing to the development of disease (Wright, Mathee & Garland 2014). Considering the recent severe Listeriosis outbreak and the proposed approach (National Health Insurance) to health care in the country (Chersich et al. 2018; Robertson 2016), the public health system will benefit immensely from a well-functional environmental health system ensuring the elimination of preventable conditions (such as FBD). This will ensure that these conditions do not overburden the public health system and its funds.

## Limitations of the study

One of the limitations of the study was that it was conducted in one municipality, and the sample size was small. The results cannot be generalised for the whole country. It is recommended that a similar study be conducted amongst EHPs from different provinces. However, it is important to consider that the Gauteng province is the most populated province in South Africa. Secondly, data were collected using a self-administered questionnaire, which could have introduced recall bias. While the study had its limitations, it can be used as a baseline to evaluate environmental or MHS, in particular, FBD outbreak investigation. Hence, the study findings contribute to the current body of knowledge and the significance of improving EHPs practices, especially with regard to the investigations of FBDs outbreaks. This will ensure that future outbreaks are adequately investigated, and that the future occurrence thereof can be prevented or limited.

## Recommendations

The study recommends the development of an educational training programme to address the insufficient knowledge, as well as developing guidelines or SOP that will assist EHPs to prepare and conduct outbreak investigations. This will also address the concerns about non-standardised notification systems and improper and non-uniform reporting. The study can build on the positive attitude of participants through introducing specialisation. Lastly, the relevant municipality needs to ensure that EHPs responsible for outbreak investigations are provided with sufficient support from management and adequate resources.

## Conclusion

The data in this study suggest that EHPs are not well conversant on how to conduct food-borne outbreak investigations. The current practices were not uniform and comprehensive. However, most participants recognise that there can be improvements in the current practices. Furthermore, the attitudes of participants were positive, even though there were concerning areas where participants were undecided.
